# Demographic risk factors of pro-inflammatory diet: a narrative review

**DOI:** 10.3389/fnut.2024.1448806

**Published:** 2024-10-17

**Authors:** Hossein Pourmontaseri, Shaghayegh Khanmohammadi

**Affiliations:** ^1^Universal Scientific Education and Research Network (USERN), Tehran, Iran; ^2^Student Research Committee, Fasa University of Medical Sciences, Fasa, Iran; ^3^Research Center for Immunodeficiencies, Children’s Medical Center, Tehran University of Medical Sciences, Tehran, Iran; ^4^Network of Immunity in Infection, Malignancy and Autoimmunity (NIIMA), Universal Scientific Education and Research Network (USERN), Tehran, Iran

**Keywords:** energy-adjusted dietary inflammatory index, E-DII, prevention, diet, demographic features, pro-inflammatory diet

## Abstract

While inflammation is a known beneficial mechanism, pro-inflammatory nutrients can lead to chronic inflammation. The energy-adjusted dietary inflammatory index (E-DII) has revealed positive associations with chronic inflammatory diseases. However, more evidence about the demographic risk factors for high E-DII is needed. Therefore, the present study reviewed the high-risk groups of people for high E-DII scores. Men had higher E-DII than women worldwide, which could be explained by the craving for energy induced by stress and higher physical activity. However, in some societies, women had higher consumption of a pro-inflammatory diet, which could be induced by compulsive eating and craving for more sweets and carbohydrates during menstruation and also can be seen among women with premenopausal syndrome. The pro-inflammatory diets were more common among elders in southern America, East Asia, and Arab countries, while some other studies had contradictory results. The proliferation of unhealthy foods, such as fast food and Western dietary patterns enriched with a pro-inflammatory diet, increased youth’s E-DII and decreased the healthy eating index among older people. Also, smokers and alcoholics tended to consume a diet with a higher E-DII, which should be investigated in further studies. Black people consumed the most pro-inflammatory diets compared with White people, especially in pregnant women. Education had a negative association with E-DII, while socioeconomic status was positively associated with a pro-inflammatory diet. Therefore, E-DII consumption had no association with access to healthy foods but is more associated with knowledge and cultural dietary habits. Moreover, further nutritional interventions are required to educate the vulnerable populations and also provide better availability of healthy food enriched with anti-inflammatory nutrients in the future.

## 1 Introduction

Inflammation is one of the most essential processes in the immune system, which responds to triggers such as injury and infection (1). While acute inflammation contributes to healing injuries and fighting pathogenic microbes, chronic inflammation causes tissue damage and other disorders, such as cancers, diabetes, autoimmune diseases, and cardiovascular diseases (2). The escape of T cells, especially CD8+, from tolerance is the primary mechanism of chronic inflammation initiation (3). Secretion of transforming growth factor-beta and the dysfunction of regulatory T cells trigger further pathways (4–6). Consequently, increased plasma levels of interferons and interleukin-1 (IL-1) and -6 (IL-6) leads to the development of chronic inflammation and result in chronic diseases (4).

Diet has a modest effect on inflammation through the regulation of plasma inflammatory factors, such as IL-6, tumor necrosis factor-alpha (TNF-*α*), and C-reactive protein (CRP) (7). Recent studies suggested the important role of obesity in the association of diet with chronic inflammation and its related metabolic diseases (8). It has been confirmed that a high-caloric diet and consuming pro-inflammatory nutrients increase fat storage in adipose tissue and result in obesity. The hyperplasia and hypertrophy of adipocytes induced by a high-caloric and -fat diet would increase cellular stress, apoptosis, and oxidative stress (9). Production of reactive oxygen species (ROS) in adipose tissue, induced by a pro-inflammatory diet, increases the IL-6 and CRP (10). Excessive intake of energy and fat causes higher secretion of leptin and other adipokines, which increase pro-inflammatory biomarkers. Also, higher energy intake and consuming fat and carbohydrates directly stimulate adipose tissue to produce IL-6, TNF-*α*, and other pro-inflammatory biomarkers (11). Intake of carbohydrates increases insulin secretion and stimulates the expression of IL-6 from adipose tissue (12). Therefore, while consuming foods enriched with fruits, fibers, and vegetables had anti-inflammatory properties, higher energy, carbohydrate, and saturated fats increase chronic inflammation (13). Also, high fat intake causes insulin resistance and increases the plasma level of TNF-*α* (14). Healthy dietary patterns, especially the Mediterranean diet, indicate cardiovascular protective effects by decreasing inflammation and thrombosis (15). Moreover, dietary patterns are associated with the development of different diseases, such as cancer, cardiovascular disease, and obesity, and can influence the outcomes of immunotherapy by regulating gut microbiome composition (16–18).

Following an exhaustive review during the 2000s, a comprehensive index was developed to assess the inflammatory potential of diet patterns for the first time (19). This index, the Dietary Inflammatory Index (DII), was eventually published by Shivappa et al. (20). A higher DII score shows a more pro-inflammatory diet, while a more negative DII represents a more anti-inflammatory diet. Using DII in nutrition studies provides a comprehensive perspective on the inflammatory potential of diet (20). In a few years, DII was rapidly applied in multiple studies worldwide. However, the revision continued, and energy-adjusted DII, also named E-DII, was developed to adjust the proportion of energy intake in assessing the inflammatory potential of diet and reveal the effect of other dietary components compared with DII. E-DII is a more global assessment of dietary inflammatory potential, which fosters a sense of connection and allows us to compare the findings in different studies worldwide (21).

To date, several recent studies showed that E-DII had a tight, significant association with different diseases, such as cardiovascular diseases, metabolic syndrome, and diabetes (22, 23). However, to our knowledge, there is a need for more evidence regarding the risk factors of potent inflammatory diets. For instance, while the proportion of dietary carbohydrates increases with aging, energy intake is significantly decreased in older people (24). Furthermore, social determinants, such as low income, also affect people’s diets; lower-income people are likelier to consume unhealthy diets (25). Therefore, the present study aimed to review different determinants of E-DII, shedding light on the factors that contribute to the inflammatory potential of an individual’s diet and their implications for chronic disease prevention and management.

The present study reviewed the studies that compared the E-DII score among different groups of demographic features to investigate the groups that are possibly susceptible to consumption of pro-inflammatory diet vulnerable to further diseases. The high-quality observational papers that studied the E-DII were included in the present study to investigate the pro- and anti-inflammatory intake compared among different groups of demographic risk factors addressed in their findings.

## 2 Search strategy

Appropriate related keywords, including pro-inflammatory diet, anti-inflammatory diet, and dietary inflammatory index, energy-adjusted dietary inflammatory index, were searched in electronic databases of Google Scholar, Scopus, Web of Science, and PubMed. We extracted searched articles from inception until October 2023. Medical Subject Headings (MeSH) terms were included. Endnote v.20 was applied to remove duplicated studies and include the full text of relevant articles. Finally, the remaining 34 articles that addressed the comparison of E-DII between demographic risk factor groups were reviewed in our study.

## 3 Determinants of E-DII

Different risk factors, such as age, sex, marital status, race, education level, socio-economic status, and addiction, have been associated with E-DII (Table 1). Each risk factor has been comprehensively discussed below:

**Table 1:** Demographic risk factors for high Energy-intake Dietary Inflammatory Index (E-DII), controversies and recommendations.

Variables	Positive relation	Negative relation	Recommendations
Age	Age and pro-inflammatory diet had a positive significant association in East Asia (36), Arab countries (43, 44), and Southern America (37). Some studies showed that pregnant women in Europe and Japan had higher E-DII (39, 40).	Most studies showed older adults had lower E-DII scores (17, 33, 34).	Further investigations must address Northern America’s controversial association between age and E-DII. Dietary interventions for high-risk older people in southern America, East Asia, and Arab countries are also required.
Gender	Most studies addressed men had higher E-DII than women in Asia, Africa, and American studies (36, 46, 56, 57).	Some controversies have been observed in American studies indicating that American women would have higher E-DII than men (17, 63).	Since most studies agree that men consume more pro-inflammatory diets worldwide, it is essential to investigate the reasons and conduct dietary interventions. Moreover, further studies are required to address the controversies of American studies about the association of sex and E-DII.
Race	Consumption of high E-DII is dramatically higher in Black individuals than in White people, especially in Northern America (34, 35, 70).	Southern American Hispanics (33) and native people in Caledonia (72) had the most anti-inflammatory diets than White people.	These findings indicated that dietary culture is the main reason for higher E-DII in Black people. Getting inspiration from Hispanics and South Americans to achieve lower E-DII would be a promising nutritional intervention.
Marital status	being single (whether divorced or widow) could be a risk factor for a pro-inflammatory diet in postmenopausal women (36, 38).	Some studies showed that married women consume more pro-inflammatory nutrients than single women (43, 45, 78).	The findings showed that although married individuals had a lower risk for pro-inflammatory diets, married older women consume higher E-DII. Therefore, further studies are required to address this controversy.
Education and socio-economic status	Most studies have associated Higher educational levels with low E-DII (38, 80, 81).	Studies in Asia and America showed that higher income would increase the consumption of pro-inflammatory nutrients (44, 45, 57).	These findings showed that while socio-economic status would be a risk factor for all individuals, having a higher educational level decreased the mean of E-DII. Knowledge and cultural properties would be more important than higher income or occupational status in having a healthy, pro-inflammatory diet.
Addiction	Smoking and Alcohol consumption were significantly association with pro-inflammatory diets (33, 59, 87).	No negative association was found.	These findings show the importance of addictions in dietary patterns. Therefore, further studies are required to investigate the mechanism of its effect on diet.

### 3.1 Age

Aging is one of the most critical determinants of health and dietary condition (26, 27). The demand for nutrients and energy changes significantly across aging. Decreased physical activity in older people causes physiological anorexia and lower energy intake (28). Also, since older people are vulnerable to morbidities, they require higher amounts of nutrients to achieve the same health condition as their younger ages; however, physiological anorexia associated with aging may prevent proper supplementation (29). Therefore, a healthy diet enriched with anti-inflammatory nutrients for aged people would guarantee their health and prevent further morbidities (30). Consuming anti-inflammatory micronutrients, especially in the elderly, modulates gene expression and inhibits signaling pathways of pro-inflammatory biomarkers such as CRP, IL-6, and TNF-*α*, which results in lower chronic inflammation and further common diseases of old age (31, 32).

A wide diversity has been observed in the association of age with E-DII, but fortunately, most studies indicated that older people had anti-inflammatory diets (17, 33, 34). However, some others showed a significant increase in E-DII among elders (35). A study on more than 142,000 Korean people showed that the mean age of individuals with a pro-inflammatory diet was dramatically higher (36). Another study on the general population of Brazil, which included 34,000 participants, showed that adults had higher E-DII than seniors (37). Moreover, women also showed varying trends in E-DII across different ages. A post-menopausal women study revealed that the E-DII scores decreased in older participants (38). Also, studies on European and Japanese pregnant women showed the same trend (39, 40); however, McCullough et al. (41) showed that older pregnant women were more likely to consume pro-inflammatory diets. Over the past decades, poor diet quality has been extended among the elderly population because of worsening social, economic, and environmental factors (42).

Interestingly, the findings in Western regions of Asia were controversial. While the studies in Arab countries showed a positive association between age and E-DII (43, 44), the older Persian people had been consuming an anti-inflammatory diet (45, 46). This controversy could be explained by the differences in the geographical and cultural varieties observed in dietary patterns in previous studies (47–49). Eventually, findings of these studies showed that pro-inflammatory diets are more common among elders in southern America, East Asia, and Arab countries. The development of cheap fast foods among different societies, especially among low socioeconomic populations, increased unhealthy eating scores. The trend in eating habits and dietary patterns has changed in the last decades, and Western Dietary patterns have become the most common (50). An unhealthy diet enriched with pro-inflammatory nutrients such as unsaturated fatty acids increases the E-DII score and develops chronic inflammation and further diseases (51). However, further studies are required to address the rationale for these differences and conduct dietary interventions to prevent complications of high E-DII observed in these societies.

### 3.2 Sex

Sex is a primary risk factor for several pathologic conditions (27). Existing evidence indicates that women and men have differences in lifestyle aspects, such as physical activity, occupation, energy, and nutrient intake (52–54). Therefore, these differences influence the consumed diet and demands for supplementations (55).

The pro-inflammatory diet was more common among men than women in most previous studies, especially in Asian and African regions (36, 46, 56, 57). Consequently, the worldwide trend of dietary patterns has increased the pro-inflammatory properties of men’s diets in different regions (58, 59). Men have higher physical activity and spend most of their time on occupational duties (52). Therefore, it is expected to observe that men ignore healthy nutrition (28). On the other hand, daily stresses increase cortisol secretion and improve people’s appetites for consuming more fat and sweets. Therefore, higher energy and carbohydrate intake increases the amount of pro-inflammatory biomarkers in plasma (60). Since men are more vulnerable to cardiovascular disease and its mortality due to their sex hormone differences than women, extended dietary interventions are required to decrease the E-DII score of men’s diet (61).

The studies in Northern America had the highest controversies. The increasing desire of American men and women of different age groups for unhealthy snacks instead of complete and healthy meals has become a significant concern (62). The increased uncontrolled sugar and energy intake has significant pro-inflammatory effects (62). While the studies by Liang et al. (17), Huang et al. (35), and Li et al. (63) indicated that American women significantly had higher E-DII than men, other studies in Northern America revealed that men were more exposed to high pro-inflammatory potent nutrition. Compulsive eating before menstruation significantly increases the energy intake without enough physical activity. Women with premenopausal syndrome (PMS) have more attraction to sweets due to fluctuations in estrogen and progesterone (64). Moreover, craving for sweets during menstruation increases the intake of energy, carbohydrates, and fats, resulting in higher E-DII (65). Therefore, these conditions could provide an appropriate explanation for the higher E-DII among women than men in the mentioned studies. However, future studies should compare the lifestyle differences between women in America and other countries to explain the reason for this controversy.

### 3.3 Race

Food culture is unique for each race (66, 67). The races found their way to achieve healthy diets. But in recent decades, the diet has significantly changed worldwide, and consumption of unhealthy diets has been growing due to modern lifestyle (66, 68).

The difference in E-DII between different races has been documented in American studies; however, there is a lack of evidence in other regions. Although existing evidence revealed that White people are more likely to consume anti-inflammatory diets (33–35, 63), some studies showed significant controversial findings (17, 38, 69). Most studies indicated that Black people had a high level of E-DII (34, 35, 70). Also, Chen et al. (38) showed that Black pregnant women dangerously are more likely to consume pro-inflammatory diets associated with poorer delivery outcomes for mothers and neonates. Previous findings showed that Black people were significantly more vulnerable to food insecurity than White people [2021, (71)]. Therefore, further dietary interventions are vital to control Black people’s unhealthy diets and restrict the inflammatory potential of their diet.

The available studies revealed that other racial minorities in North and South America, especially Hispanics, widely use anti-inflammatory diets (33). Moreover, Hispanic pregnant women had healthy anti-inflammatory nutrition during pregnancy (39, 41). The study conducted by Paquet et al. (72) showed that the native people of New Caledonia, Melanesians, are interested in anti-inflammatory foods. This study also showed that Melanesians had a better health status than Europeans (tending to be closer to the Western food pattern) (72). Consequently, it would be concluded that the prevalence of pro-inflammatory diets in Black people is more related to food culture than access to food, literacy level, and inequalities (73, 74).

The study by Masaad et al. (44) showed that Arab people had slightly attended to pro-inflammatory diets than non-Arab people living in the Middle East. On the other hand, Chinese people had significantly lower E-DII scores than the different races, such as Malay and Indian, in East Asia (57). However, there needs to be more evidence addressing the differences between races living around the world comprehensively. The findings of our review on race differences by E-DII suggest that expanding the food culture of races that have an anti-inflammatory diet among races, such as Black and Arab people, would be a promising way to prevent the incidence of diseases related to chronic inflammation in the future.

### 3.4 Marital status

The role of marital status in dietary intake has been well-known during the last decades (75–77). While Sreeja et al. (36) indicated that married Korean people had lower E-DII scores than single individuals, more prevalence of a pro-inflammatory diet was observed in married American individuals (17). Small sample-sized studies on female participants showed that married women consumed pro-inflammatory diets (43, 45, 78). In contrast, Chen et al. (38) showed that a high E-DII score is more observed in single postmenopausal women than married women. This study also showed that age had a significant positive association with higher E-DII scores in women, as mentioned before (38). Therefore, being single (whether divorced or widow) could be a risk factor for a pro-inflammatory diet in postmenopausal women.

### 3.5 Education and occupation

Educational and occupational levels are important determinants of healthy eating and lifestyle in all societies (79). In East and West Asia, most evidence showed that individuals with higher educational levels had significantly lower E-DII scores (38, 80, 81). In contrast, the study conducted by Asgari et al. (45) revealed that obese women with higher educational levels had a more pro-inflammatory diet than low-educated people. Interestingly, this study also showed that the participants with higher occupational levels had more E-DII scores than low-income individuals (45). Moreover, other Asian studies showed that higher income and occupational level were tightly associated with a pro-inflammatory diet (44, 57). Healthy eating is an essential aspect of lifestyle for pregnant women and has a vital role in their children’s nutrition (82). Therefore, Asian studies showed that a higher educational level would help decrease E-DII score, while higher occupational level and income provide the conditions for a pro-inflammatory diet.

There is significant controversy in American studies regarding the association of education with E-DII (33, 34, 63, 69). Individuals who were educated in college or higher consumed pro-inflammatory diets, while some studies show that people with low educational levels (less than 9 years of education) obey more anti-inflammatory diets (37). Pereira et al. also suggest that people with higher incomes consume pro-inflammatory nutrients (37). Therefore, while the educational level is closely associated with higher E-DII scores, studies have shown that a higher educational-occupational level increases the pro-inflammatory potential of diet. Also, previous studies showed that both higher education and socioeconomic status develop healthy eating behaviors among different populations, but socioeconomic status is more important than education (83). While educational interventions increased the consumption of whole grains, vegetables, and fruits, recent observations represented that people with inadequate financial resources crave more energy than healthy eating, even if they are well-educated (84, 85). Therefore, although education is a basic factor of healthy eating, financial barriers can influence people to consume more unhealthy, pro-inflammatory nutrients enriched with fat, energy, and carbohydrates.

### 3.6 Addiction

Although addiction is not a demographic risk factor, it is closely associated with demographic factors such as age, sex, ethnicity, and socioeconomic status (86); thus, we decided to discuss the association of addiction with the E-DII level.

Approximately all existing findings confirmed that alcoholics and smokers are interested in dramatically inflammatory potent nutrients (33, 59, 87). Some studies revealed that a pro-inflammatory diet is more prevalent in smokers up to 3 or 4 times compared with non-smokers (17, 36). Smoking not only decreased the appetite of adults but also had a significant association with unhealthy diet in previous studies (88). Moreover, Chao et al. indicated that dietary intervention and expanding healthy eating habits would be more difficult for smokers (89).

The mechanisms of the association between addiction and unhealthy eating habits, especially higher E-DII, are still unclear. However, the activation of reward centers in the brain could be a responsible mechanism that increases the craving of addicted people for more sweets, carbohydrates, and energy (90). Also, recent investigations showed that addicts experience urge, hunger, and compulsive eating behaviors (91). Consequently, smoking and alcohol are two important neglected risk factors of the inflammatory diet, which the majority of studies have shown can be closely related to unhealthy eating habits. Also, the use of drugs, such as narcotics, cannabis, and modern psychedelics, are other substances whose addiction can be closely related to the desire for inflammatory diets, and there is limited information about them till now. On the other hand, addiction to several materials, such as coffee or tea, would be another risk factor for higher E-DII that would be addressed in further studies.

## 4 Conclusion

The present study reviewed the papers addressing the demographic risk factors for a pro-inflammatory diet. Although addiction, low educational level, high income, and being single were common risk factors for high E-DII scores, there were some significant controversies regarding sex and race. Several controversies observed in previous studies about age, gender, and race (in some cases) would be required to be investigated in future studies to achieve more precise findings. The high prevalence of pro-inflammatory diets in Arab and Black people could be a hazard to the future of healthy eating worldwide. Most of the existing evidence was analyzed by unadjusted models. Therefore, further studies are required to address the demographic risk factors of high E-DII and provide promising dietary interventions for vulnerable populations. Moreover, nutritional interventions, educating vulnerable populations, and ameliorating their availability of healthy food enriched with anti-inflammatory micronutrients would help to alleviate their dietary habits and prevent further chronic inflammatory diseases.
